# Symptomatic Infundibulopelvic Dysgenesis in an Adolescent

**DOI:** 10.1155/2015/307319

**Published:** 2015-04-09

**Authors:** Daniel Pitts, David Chalmers, Brian Jumper

**Affiliations:** ^1^Tufts University School of Medicine, Boston, MA 02111, USA; ^2^Department of Urology, Maine Medical Center, Portland, ME 04102, USA

## Abstract

Infundibulopelvic dysgenesis is a rare condition characterized by congenital malformation of the pelvicalyceal system. We present the case of an 18-year-old boy with chronic intermittent right flank pain and cystic dilation with parenchymal thinning on ultrasonography. The left kidney was normal. The patient denied dysuria, constipation, and history of UTIs or renal calculi. Cystoscopy with retrograde pyelogram showed marked stenosis of the right pelvicalyceal system and anatomy unfavorable to stenting. The patient's symptoms were unresponsive to conservative management. Reconstruction of the right collecting system was unsuccessful and a simple nephrectomy was performed, which led to complete resolution of his symptoms.

## 1. Introduction

Infundibulopelvic dysgenesis is a term used to describe one condition amongst a spectrum of congenital disorders of the pelvicalyceal system. This spectrum includes focal and multifocal abnormalities leading to multicystic dysplastic kidney (MCDK), infundibulopelvic stenosis, calyceal diverticula, and ureteropelvic junction obstruction [1]. Infundibular stenosis has been associated with MCDK; in one series 2.5% of children with MCDK have some degree of stenosis in the contralateral kidney [2].

We report the case of an 18-year-old boy with episodic flank pain that was poorly controlled with conservative management. We discuss our decision-making, surgical approach, and the challenges associated with multifocal anatomical changes.

## 2. Case Report

**Figure 1:**
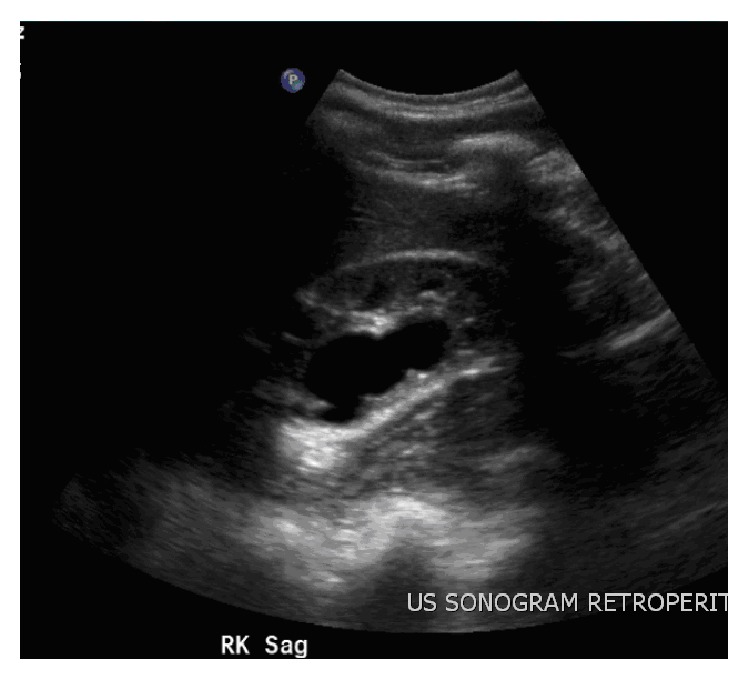
Ultrasound of right kidney read as pelviectasis and possible renal cysts.

An 18-year-old boy presented to his pediatrician with right upper quadrant pain of one year duration. Initial workup included a normal esophagogastroduodenoscopy and urinalysis which showed calcium oxalate crystals, prompting referral to pediatric nephrology. Subsequent renal and bladder ultrasound showed cystic spaces and cortical thinning in the lower pole of the right kidney (Figure 1). The upper pole was relatively spared. The left kidney, bladder, and ureters appeared normal. This was interpreted as pelviectasis with possible parenchymal cysts and prompted referral to pediatric urology.

The patient reported eighteen months of persistent discomfort and a sensation of fullness in his right flank. The pain acutely worsened about once per month and was exacerbated by large volumes of fluid intake. The pain was never on the left side and was not associated with hematuria, dysuria, urinary retention, or cloudy urine. The patient denied fevers or chills and had no history of UTIs. He denied nausea, vomiting, diarrhea, and constipation. The pain was unresponsive to ibuprofen. The patient was a high-school athlete in overall good health. Family history was negative for any genitourinary anomalies.

On examination the patient appeared well, in no apparent distress. He was afebrile and normotensive. His abdomen was soft, nontender, and nondistended with no guarding and no hepatosplenomegaly or masses. The remainder of his exam was unremarkable.

Radioisotope renography with technetium99m-mercaptoacetyltriglycine (Tc99m-MAG3) with Lasix washout demonstrated accumulation of radiotracer activity within a dilated pelvicalyceal system in the lower pole of the right kidney, with delayed flow, uptake, and excretion. There was normal flow and slightly delayed uptake and excretion in the upper pole. Post-Lasix t1/2 was greater than 24 minutes. The left kidney was normal and without obstruction. Calculated renal function was 61.5% to the left kidney and 38.5% to the right kidney.

The differential diagnosis at this point included an obstructive process within a duplex system such as ureteropelvic junction obstruction or calyceal diverticulum, cystic kidney disease, malignancy, or other congenital abnormalities of the kidney.

**Figure 2:**
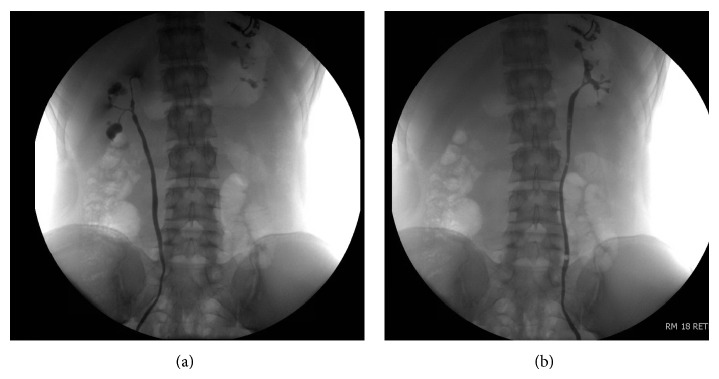
Retrograde pyelogram of right kidney (a) showing stenotic infundibulae, the absence of a renal pelvis, and dilated calyces. Left kidney (b) has a normal collecting system.

To assess the etiology and potentially place a ureteral stent to alleviate symptoms, cystoscopy with retrograde pyelogram was performed. This demonstrated calyceal diverticula and infundibular stenosis in the right kidney (Figure 2). The anatomy was not amenable to stenting.

**Figure 3:**
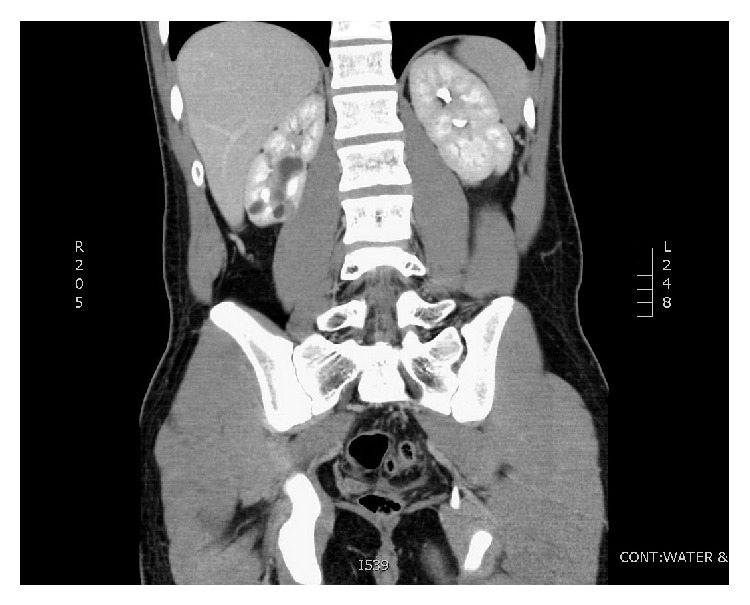
CT scan with contrast showing dilated calyces and associated cortical thinning in the lower pole of the right kidney. The left kidney appears normal.

Throughout the workup, ibuprofen and fluid limitation had provided unsatisfactory pain control. Options for surgical intervention included stenting, a percutaneous approach such as endoscopic dilation or infundibulotomy to improve drainage, pyeloplasty, or partial nephrectomy. Given the multifocal nature of stenosis, a single stent would have been inadequate and infundibulotomy would not have been feasible. Pyeloplasty was inappropriate as there was no renal pelvis to reconstruct (Figure 2). Given the normal function of the contralateral kidney and the preserved function in the upper pole of the involved kidney, the decision was made to perform a partial nephrectomy to remove the cystic dilations while sparing as much renal parenchyma as possible. Computed tomography (CT) was performed to plan the surgical approach and showed dilated calyces and diffuse cortical thinning in the lower pole of the right kidney (Figure 3). The upper pole of the right kidney was normal. The imaging showed no evidence of ureteropelvic junction obstruction. The multifocal nature of the dilated calyces ruled out a single calyceal diverticulum. Delayed images demonstrated dependently layering contrast, ruling out cystic dysplasia. Contrast was excreted by a single ureter, ruling out a duplex collecting system.

The patient was taken to the operating room with the intent to perform a partial nephrectomy of the obstructed, symptomatic calyces in the right lower pole. Open and robotic approaches were considered. We were concerned that defining the surgical margins and reconstructing a ureteropelvic junction would be challenging; therefore we elected for an anterior subcostal open approach to optimize exposure.

In the operating room the hilar structures were identified and intraoperative ultrasound identified dilated calyces extending 2/3 of the way up the posterior aspect of the kidney. This was more extensive than suspected based on preoperative imaging. The renal artery and vein were clamped and the cystic areas were excised. Less than 1/3 of the kidney remained, rendering reconstruction unfeasible. In light of these findings and the symptomatic nature of the obstruction, a total nephrectomy was performed. The patient's postoperative recovery was uneventful and follow-up confirmed his previous right flank pain was completely resolved.

## 3. Comment

Infundibulopelvic dysgenesis refers to a spectrum of disorders of development of the pelvicalyceal system. It can present in various ways, including recurrent urinary tract infections, hypertension, proteinuria, and headaches [3]. Malformations that cause stenosis manifest as obstructive symptoms, calyceal dilatation, and the appearance of hydronephrosis on imaging.

To date, the largest case series regarding infundibulopelvic dysgenesis was published by Husmann et al. in 1994 [4]. This series included 21 patients and reported that 90% of patients had some measure of bilateral involvement. When these patients underwent nephrectomy for progressive hydronephrosis, surgical pathology showed hyperfiltration injury. An additional study by Dally et al. described two hundred children with MCDK, five of whom had infundibular stenosis in the contralateral kidney [2]. Of these, four presented as neonates with stenosis seen by ultrasound or noted as an asymptomatic palpable mass. The index case was a 16-year-old who presented with flank pain, similar to our patient. It is unclear whether these presentations represent different disease processes, congenital stenosis or stenosis that progresses over time.

It has been theorized that infundibulopelvic dysgenesis is the result of early or late budding of the ureter during embryogenesis [5, 6]. During normal development of the metanephros, glial cell line-derived neurotrophic factor (GDNF), a peptide secreted by the metanephric mesenchyme, stimulates and localizes outgrowth of the ureteric bud via activation of the RET receptor. Numerous other factors, including PAX2, Eya1, and FoxC1/C2, positively and negatively regulate the expression of GDNF, thereby guiding development of the ureteric bud [7].

Many of the factors regulating ureteric bud outgrowth also stimulate branching and dilatation to form the renal collecting system. PAX2 and vitamin A are transcription factors that induce branching of the ureteric bud. Meanwhile, both the* Emx2* and* Sall1* genes seem to be necessary for stimulating branching and dilation of ureteric buds. The absence of any of these stimulatory factors, or excess of inhibitory factors, could lead to a scenario where the ureteric bud does not branch and dilate appropriately, causing stenosis of the calyceal system. The earlier the stage at which these imbalances occur, the more significant the resulting dysgenesis is likely to be. Given the significant stenosis and lack of calyceal branching observed in our patient, development of the collecting system likely failed at a relatively early stage. The unilateral nature of this process speaks against a complete genetic defect, although mosaicism is also possible.

The proper management of patients with infundibulopelvic stenosis has been discussed in the literature [4]. Typically, renal function is monitored, while giving ACE inhibitors to protect renal function. Surgical intervention is reserved for patients with pain from obstruction, progressive hydronephrosis, and symptomatic stone disease proximal to the stenotic infundibulum.

It is important to recognize how management decisions would change in the setting of a solitary kidney or bilateral disease. Our patient was relatively unique in that he only had unilateral disease leading to caliectasis and pain. We felt that a partial nephrectomy was appropriate in hopes of removing the affected and symptomatic portion of the right kidney. This decision put the normal upper pole at risk and led to the unintended outcome of a simple nephrectomy. This outcome was mitigated by the normal contralateral kidney; however this relatively aggressive management decision would not have been made in the presence of bilateral disease or a solitary kidney. We would recommend conservative management in these situations, with either additional pain control, endoscopic dilation, or infundibulotomy, stenting of the stenotic segments, or percutaneous nephrostomy tubes. In patients with bilateral infundibulopelvic stenosis, partial nephrectomy may not be a valid option for treatment, given the difficulties associated with reconstruction of complex anatomy. In such patients all other options should be considered.
